# Efficiency of Experimental Formulation Containing *Duddingtonia flagrans* and *Pochonia chlamydosporia* against *Moniezia expansa* Eggs

**DOI:** 10.3390/pathogens12081028

**Published:** 2023-08-10

**Authors:** Giancarlo Bomfim Ribeiro, Ially de Almeida Moura, André Ricardo e Silva, Jackson Victor de Araújo, Caio Márcio de Oliveira Monteiro, Júlia dos Santos Fonseca, Ana Patrícia David de Oliveira, Wendell Marcelo de Souza Perinotto

**Affiliations:** 1 Programa de Pós-Graduação Integrado em Zootecnia, Centro de Ciências Agrárias, Ambientais e Biológicas, Universidade Federal do Recôncavo da Bahia, Cruz das Almas 44380-000, BA, Brazil; giancarlo.bomfim@gmail.com; 2 Programa de Pós-Graduação em Ciência Animal, Universidade Estadual de Santa Cruz, Ilhéus 45662-900, BA, Brazil; ially20@hotmail.com; 3 Departamento de Veterinária, UFV, Universidade Federal de Viçosa, Viçosa 36570-900, MG, Brazil; andre.silva@cinergis.com.br (A.R.e.S.); jvictor@ufv.br (J.V.d.A.); 4 Departamento de Biociências e Tecnologia, Instituto de Patologia Tropical e Saúde Pública, Universidade Federal de Goiás, Goiânia 74605-050, GO, Brazil; caiosat@gmail.com; 5 Programa de Pós-Graduação em Ciências Veterinárias, Departamento de Epidemiologia e Saúde Pública, Universidade Federal Rural do Rio de Janeiro, Seropédica 23897-000, RJ, Brazil; julia.d.fonseca@ufv.br; 6 Instituto Federal de Educação Ciência e Tecnologia, Salvador 40301-015, BA, Brazil; anapatriciatn@hotmail.com

**Keywords:** biological control, helminthophagous fungi, tapeworms

## Abstract

This study aimed to evaluate the effectiveness of the experimental formulation containing chlamydospores of *Duddingtonia flagrans* and *Pochonia chlamydosporia* fungi, against *Moniezia expansa*. Two experiments were carried out. The first experiment evaluated the in vitro efficacy using 1 g of the experimental formulation (V1) added to 100 *M. expansa* eggs and the control (V2) (without the fungal formulation). Intact eggs or eggs with alterations were counted in order to evaluate their effectiveness. The second experiment evaluated the action of the fungal formulation on *M. expansa* eggs after passing through the gastrointestinal tract of goats. Three groups were identified as B1, B2, and B3, which received 1.0, 1.5 g of experimental fungal formulation, and placebo, respectively. In experiment 1, all the eggs in V1 were subjected to the predatory action of fungi, while in V2, the eggs remained intact. In experiment 2, the reduction of eggs in groups B1 and B2 were 49% and 57% 24 h after ingestion, 60% and 63% 48 h after, and 48% and 58% 72 h after. The predatory capacity against *M. expansa* eggs shown in the tests demonstrated that experimental fungal formulation has the potential to be used on integrated helminth control programs.

## 1. Introduction

Monieziosis is a disease caused by tapeworms from the genus *Moniezia* sp. The species *Moniezia expansa* is the most important for small ruminants within the genus, as it parasitizes the small intestine of these animals [1,2]. Adult animals are usually asymptomatic, but the young end up being the most affected, compromising growth and productivity [3,4]. Eventually, when infections involve numerous parasites, they can cause severe damage to the small intestine, constipation, intestinal obstruction, and death of animals, causing direct economic losses [5].

Anthelmintic resistance, common to all classes of helminths, is a global challenge [6]. Associated with the limitation of active principles acting against tapeworms, it can compromise the efficiency of parasite control [7,8,9]. These limitations pave the way for the development of new technologies that can be integrated into current breeding systems without compromising productivity and meeting the need for rational use of anthelmint products [10].

Biological control is one of the options to complement helminth control actions. Helminthophagous fungi have been shown to be effective in eliminating the free-living stages of helminths in pastures, decontaminating the environment, and reducing consequently the reinfection of animals [11,12,13]. These fungi act during the free-living stages of helminths [14] and exert their predatory action through different mechanisms of action, capturing and destroying the parasites [15]. *Duddingtonia flagrans* is known to produce a three-dimensional adhesive network responsible for capturing and eliminating nematodes [16].

*Duddingtonia flagrans* has been widely studied, and there are already commercial products which allow the implementation of biological control for parasites in animal production [12,17,18,19]. The association between different species of fungi is important to expand the spectrum of action of fungal formulations, especially if the fungi act on different targets of the life cycle of the parasites [20].

Previous studies involving *Pochonia chlamydosporia* proved its in vitro and in vivo ability to control helminths that affect domestic animals [20]. There is a classification system [21] that defines three types of ovicidal activity: type 1–The egg is not penetrated, but development ceases or causes the development of anomalous larvae; type 2–The hypha does not penetrate the egg, but both the shell and the embryo are enzymatically damaged; type 3–The hypha penetrates the egg, colonizing the interior of the egg and the embryo.

Its ovicidal activity on *Taenia saginata* eggs has already been documented [22]. As with the previous cestode, *M. expansa* needs intermediate hosts, and its eggs remain in the environment until ingested [23,24]. Thus, their eggs can be a suitable target for this fungus. Furthermore, the search for new formulations and the association of different mechanisms of action from different groups of fungi may represent advances to control these parasites [25].

Given this situation, this study was carried out to evaluate the ability of an experimental fungal formulation, containing *D. flagrans* and *P. chlamydosporia* chlamydospores, to reduce the number of *M. expansa* eggs before and after passing through the gastrointestinal tract of goats.

## 2. Materials and Methods

### 2.1. Obtaining Moniezia expansa Eggs

*Moniezia expansa* eggs were obtained by dissecting the uterus of adult parasites from the intestinal loops of goats slaughtered in the municipality of Socorro, São Paulo, Brazil, provided by the veterinary industry of the state of São Paulo.

### 2.2. Obtaining the Experimental Fungal Formulation

The experimental fungal formulation used in the study was produced and supplied by the veterinary industry of the state of São Paulo, Brazil. The formulation contained chlamydospores of *D. flagrans* and *P. chlamydosporia* at a concentration of 1.6 × 106/g of each fungus.

These microorganisms are registered at the National Genetic Heritage Management System (SISGEN), registration number A8A8866, in the research line of Biological Control of nematodes by the researcher Jackson Victor de Araújo from the Federal University of Viçosa, MG-Brazil.

### 2.3. Goats Used in the Experiment

Eighteen goats belonging to the herds of the Federal University of Recôncavo da Bahia, crossbred Saanen and Anglo-Nubian of both sexes (males and females) with ages between 6 and 18 months and weighing around 30 kg, were used. This study was approved by the Ethics Committee in the Use of Animals (CEUA) of UFRB (protocol 23007.00006228/2019-16).

### 2.4. Experimental Tests

#### 2.4.1. Experiment 1

Initially, we carried out a study to evaluate the effectiveness of the fungus before administration to the animals (without the action of the gastrointestinal tract and its development in the feces). For this purpose, 2% Agar–Water culture media were used in Petri dishes and two groups were created containing five Petri dishes each, with 100 eggs of *M. expansa* added to each plate. The groups were identified as V1 and V2. In group V1, 1 g of the fungal formulation was added, and V2 was the control without the addition of the formulation. The plates remained in a Biological Oxygen Demand (B.O.D.) incubator at 26 °C in the dark for 10 days. After this period, the integrity of the eggs was evaluated according to the parameters established by Lýsek [21].

#### 2.4.2. Experiment 2

The evaluation using animals was carried out to verify the predatory capacity of the fungus after passing through the gastrointestinal tract of goats. The animals were weighed and treated with the anthelmintic ivermectin 0.08% at a dose of 1 mL for every 4 kg orally and 0.75 mL of albendazole 5% for every 10 kg orally, following the indicated prescription in the medicine leaflet. Fifteen days after deworming and confirming the absence of helminth eggs in the feces, the animals were raffled to determine their respective groups. Three groups containing six animals were formed. The goats in group B1 received 1 g of experimental fungal formulation per animal, those in group B2 received 1.5 g of experimental fungal formulation per animal, and the goats in group B3 received placebo (1.5 g of feed per animal). The treatments were performed with a single dose; the product was mixed with the feed and offered to the animals. The supply was carried out in individual troughs, and the animals were observed and released when they ingested all the offered content to guarantee that the whole product had been consumed.

After administration, stool samples were collected from each animal directly from the rectal ampulla, weighing approximately 5 g at intervals of 24, 48, and 72 h. Each sample was homogenized and later mixed to form a common sample by group and collecting time. Then, 2 g of feces were transferred to Petri dishes containing 2% Agar–Water, using plates by group and time.

All plates were added with 100 eggs of *M. expansa*. Therefore, ten aliquots of 10 μL each were obtained from 20 mL of an aqueous suspension of *Moniezia* eggs stored in a 40 mL cell culture bottle and quantified. Then, the mean number of eggs per sample was calculated, and from this mean, the suspensions were adjusted for 100 eggs for the Petri dish. Plates were set in a B.O.D. incubator at 26 °C in the dark for 15 days. Every three days, plates were inspected to verify the presence of characteristic conidia and conidiophores of the fungus, according to the classification proposed by Hoog [26], and also to check for egg predation. The integrity of the eggs was evaluated according to the parameters established by Lýsek [21].

To obtain the reduction value percentages, the following formula was used:

Reduction % = (Average number of eggs in the control group − Average number of eggs in the treated group)/(Average number of eggs in the control group) × 100

### 2.5. Statistical Analysis

The data were submitted to the Shapiro–Wilk normality test with a 95% confidence level. The Mann–Whitney test was performed to assess whether the number of intact eggs differed from one group to the other in experiment 1. In experiment 2, the variables were subjected to tests of homogeneity of variance (Levene with a 95% confidence interval) and subsequently to analysis of variance (ANOVA). Means were compared by Tukey’s test (*p* < 0.05). To calculate the effect size, we used squared gamma (ω^2^). All analyses were performed using the R software version 4.1.0.

## 3. Results and Discussion

In experiment 1, the fungus developed in the Petri dishes of V1 and its hyphae colonized the eggs of *M. expansa*, causing structural changes in the eggs. Changes described by Lýsek [21] were observed on all eggs, and the result of the reduction test was 100%.

While evaluating the integrity of *M. expansa* eggs, the results showed that the number of eggs was lower in group V1 (Z = 2.9912, *p* = 0.0123, r = 0.86) than in V2. The median for V1 was 0, and for group V2, it was 113.

**Figure 1:**
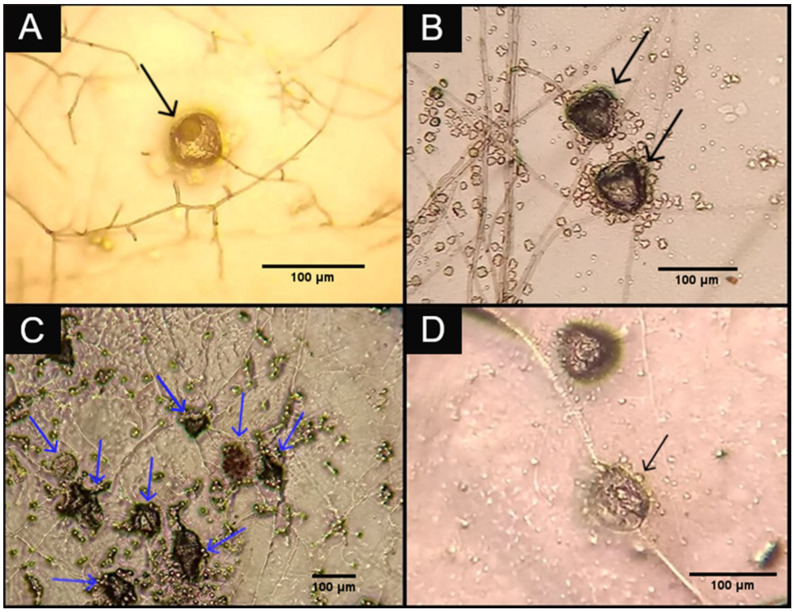
Images (**A**,**B**): Type 1 ovicidal effect on *Moniezia expansa* eggs (black arrows). Image (**C**): *Moniezia expansa* eggs whose integrity is compromised by *Pochonia chlamydosporia* and type 2 ovicidal effect (black arrows). Image (**D**): *Moniezia expansa* egg penetrated by *Pochonia chlamydosporia* hypha showing type 3 ovicidal effect (blue arrows). All images were obtained from the groups that received treatment with the experimental fungal formulation, groups B1 (total dose of 1 g of the formulation for each animal) and B2 (total dose of 1.5 g of the formulation for each animal).

In experiment 2, the development of hyphae on the culture medium in the Petri dishes of groups B1 and B2 was verified, but hyphae of *P. chlamydosporia* were not verified in group B3. The experimental fungal formulation was not offered to the animals that provided the feces used in group B3, justifying the absence of the fungus in the plates of this group. Lytic effects on the eggs occurred even after passage through the gastrointestinal tract of goats (Figure 1).

The reduction of eggs after gastrointestinal transit was evaluated, and a significant effect was observed in the 24-h treatments (F = 30.32, *p* < 0.0001, ω^2^ = 0.685); 48 h (F = 38.81, *p* < 0.0001, ω^2^ = 0.737); 72 h (F = 31.32, *p* < 0.0001, ω^2^ = 0.692). The values assigned to each treatment and collecting time are shown in Table 1.

Fungal formulations that make use of associations can obtain synergistic effects with this. Furthermore, they can broaden their spectrum of action as long as the associated fungi act on different species of helminths [27]. *D. flagrans* is a larvae-predating nematophagous fungus that produces sticky three-dimensional traps. *P. chlamydosporia* is an opportunistic and ovicidal fungus that produces a structure called the oppressor capable of penetrating eggs [12,13,16,28].

The association of *P. chlamydosporia* and *D. flagrans* probably would not generate a synergistic effect to reduce the number of eggs of *M. expansa* specifically. As a result, *D. flagrans* did not demonstrate the ability to exert a type 3 lytic effect on helminth eggs, despite having a type 1 lytic effect on *Fasciola hepatica* eggs after 21 days of experiment [29]. However, *Pochonia* sp. could collaborate with the reduction of nematode eggs and probably reduce the number of recovered lavas; since acting alone or in association with *D. flagrans* and *M. thaumasium*, it reduced the number of cyathostomine larvae by 73.2%, 86.8%, and 77.3%, respectively [30]. However, when it was used as the only agent in field evaluations, it was unable to control the free-life stages of bovine gastrointestinal nematodes [20]. Therefore, considering the reduction of eggs, it is unlikely that *D. flagrans* contributed significantly to the results presented. Thus, the experimental fungal formulation used in this work acts against larvae and eggs of helminths. For each of the objectives in the present work, one of the two fungi was predominant (*D. flagrans* and *P. chlamydosporia*). The effect against larvae occurred mainly due to the action of *D. flagrans*, which has already been studied widely [12,13,31,32]. As for the ovicidal action, the preponderant role was played by *P. chlamydosporia*, as evidenced in the present study.

The ovicidal capacity of *P. chlamydosporia* has already been described for *Dipylidium caninum* (reduction of 92.2% and 88.4% for isolates VC1 and VC4), *Taenia taeniaeformis* (73.8% of type 3 lytic effect for isolate VC4), *T. saginata* (reduction of 54.2% and 42.4% for isolates VC1 and VC4), and *Anoplocephala perfoliata* (reduction of 71.17% for isolate VC1) [33,34,35]. The results for the reduction in the number of eggs are shown in Table 2. The treatments tested in the present work were able to reduce the number of eggs at all collecting times, and the results did not differ among themselves when observing the different treatments.

The study with *Paecilomyces lilacinus* demonstrated an ovicidal effect by showing a type 3 mechanism of action on *Moniezia* sp. eggs [36]. Likewise, type 3 effect on the eggs was observed in the present study, evidencing ovicidal action. These results demonstrated the ability of the fungus to colonize and prey on eggs in large proportions. This characteristic is considered crucial for biological control agents [37]. Thus, *P. chlamydosporia* is part of the fungi which perform against this tapeworm, expanding the range of options for biological control. However, the effectiveness of *P. lilacinus* appears to be lower than that of *P. chlamydosporia*. When two fungal isolates of *Pochonia* sp. (VC1 and VC4) showed a type 3 lytic effect in 71% and 74% of *Oxyuris equi* eggs, the effects for *P. lilacinus* were significantly smaller at 54% [38]. In addition, these fungi were evaluated for eggs of *Ascaridia galli* and *Toxocara canis*. In this case, *Pochonia* sp. also demonstrated a greater reduction in the number of viable eggs (86% and 67%), contrary to the reduction provided by *Paecilomyces* sp., which was lower (29% and 28%). These values correspond to the studied parasites obtained at the end of the experiment (42 days) [39]. Therefore, these results demonstrate the superiority of *Pochonia* sp. as an agent for biological control and the importance of knowing its effectiveness in vivo studies and on other helminths of veterinary interest.

In the present study, after passing through the gastrointestinal tract, there was a predatory effect on the eggs, but this reduction was lower than that observed in experiment 1. This difference may be the result of a lower amount of chlamydospores under the conditions of experiment 2. Significant losses of chlamydospores occur during the transit through the gastrointestinal tract. The exposure of chlamydospores to the conditions of such environment can generate relative losses of 89.7% of chlamydospores [40]. Furthermore, the amount of formulation used in experiment 1 (1 g) was either identical to or very close to the amount supplied to the animals in experiment 2. In this sense, the high concentration of chlamydospores in experiment 1 would justify the destruction of all eggs in experiment 1, V1 group.

As *M. expansa* needs intermediate hosts, the main hosts are oribatid mites, and their eggs remain in the environment until they are ingested [24]. The eggs show adaptive characteristics in relation to their intermediate host, the oribatid mites, and to the environment that are essential for their survival. The eggshell provides mechanical protection from the environment. The piriform apparatus that protects the embryo in the innermost layer of the egg is composed of keratin, and its submembrane located at the interface between the two anterior layers has a lipid coating that protects the egg from dissection [41]. As their eggs remain exposed and only develop after ingestion by a host, the fungus has the opportunity to exert its lytic effects. Given this scenario, egg reduction was attributed to the effects of this fungus, being that the present study is the first report of *P. chlamydosporia* ovicidal action on *M. expansa* eggs in an in vitro test and after passing through the gastrointestinal tract of goats.

*Moniezia expansa* eggs have a thick wall that requires mechanical breakdown of the membrane and enzymatic digestion of the piriform apparatus, which protects the embryo. The shell is resistant to a variety of proteolytic enzymes, amylases, and lipases [41]. These challenges must be overcome in order for the fungus to act. In the presence of eggs, metabolites are produced, such as glycoproteins, which help in the surface adhesion process. After adhesion, appressoria are produced at the end of the germ tubes, which perform physical and biochemical penetration functions [42]. The mechanical and enzymatic actions result in the rupture of the outer layer of the egg, removing the outer proteinaceous vitelline membrane and allowing the penetration of the hyphae [43].

Proteases produced by the fungus play a relevant role in the rupture of the eggshell. The crude extract of *P. chlamydosporia* composed of proteases was used on *Ascaridia galli* eggs and showed a reduction effect of 64.1% [44]. The crude extract reduced the hatching of *Ancylostoma* sp. eggs with a reduction of 76.8% [45]. *M. expansa* eggs are rich in keratin in the composition of their structures, such as the shell and the piriform apparatus. These structures are disrupted under the effect of proteases and mechanical action, effects very similar to those observed during infection of intermediate hosts [41]. These data demonstrate the importance of this class of enzymes in the role of the fungus on parasitized eggs. It is likely that these enzymes acted on *M. expansa* eggs and helped in the penetration and colonization process.

*Pochonia chlamydosporia* produces relevant structural changes in eggs exposed to hyphae, interfering with the development and hatching of larvae [46]. Extracellular enzymes (proteases, chitinases, phosphatases, and lipases) act on the eggs, and the hyphae of the fungus colonize the host tissues [47,48]. These are the mechanisms of action involved in the parasitism of the fungus on its hosts and are probably the same ones performed on *M. expansa* eggs.

The production of resistance spores called chlamydospores enables the survival and dissemination of this fungus; this structure has important glycogen reserves and has thick walls produced along the hyphae [49,50]. Due to their characteristics, chlamydospores are able to transit through the gastrointestinal tract of ruminants and maintain their predatory capacity when they are eliminated in the feces [28,40,51]. In addition to these capabilities, the production of numerous chlamydospores is what allows the development of products for biological control [52]. The mechanisms of action and the ability to survive the gastrointestinal tract allowed the fungus to remain viable and perform against *M. expansa* eggs.

*Moniezia expansa* infections rarely result in the death of animals, but they can lead to economic losses due to reduced growth rate, in addition to other factors [3,49]. The occurrence of infections is not uncommon, and the most susceptible animals are young, possibly the most affected by complications from the disease [53]. The development of control mechanisms that may allow reduction of the infection of these animals could lead to an improvement in the productivity indexes of the herds.

As an example of performance improvement, the animals that received 1 g of *D. flagrans* chlamydospores for each 10 kg of live weight in the Bioverm^®^ commercial formulation resulted in an average weight gain of 300 kg in the treated group, against an average weight of 261 kg in the control group, leading to significant economic gains both by increasing weight gain and by reducing deworming costs [54]. The reduction of infective forms of nematodes produced a significant reduction in the OPG of the animals, and the reduction in parasitism improved the productive performance.

With the use of biological control, it becomes possible to reduce or ration the use of anthelmintics, which according to Castro [55], have been used irrationally over decades, generating an increasing resistance of parasites to the bases of chemical products available on the market. With the scarcity of bases due to the resistance generated over time, the control of helminths becomes increasingly difficult. Allied with this, according to Porto Filho [56], the residues left by the use of anthelmintics are of great importance in single health, with global health implications. These products leave residues in meat, milk, and derivatives, which reach the table directly, impacting the health of the consumer. In addition, in the application, workers are exposed to these residues due to a lack of guidance and adequate personal protective equipment (PPE). Such residues contaminate soil, water, and vegetation as a result of applications and also due to improper disposal of their packaging. Therefore, there are direct and indirect impacts on the health of the population, animal health, and environmental health, thus configuring a problem in One Health.

The proper use of anthelmintics and the reduction of their dependence have become a necessity to circumvent the consequences imposed in the current situation of anthelmintic resistance, which has been an obstacle to the control of parasites worldwide [57]. Therefore, alternative measures are imperative to control helminths that infect production animals.

In addition, the presence of residues in products of animal origin can pose a risk to public health [58]. The withdrawal period for certain anthelmintics makes their use unfeasible in certain production systems, such as dairy cattle. In some situations, there is non-compliance with the waiting period required for the proper use of the product, thus causing the presence of residues in foods of animal origin [59]. These residues have an impact on the health of herds, public health, and the environment, therefore causing a global health problem.

On the other hand, helminthophagous fungi do not cause anthelmintic resistance, do not contaminate soil and water, and do not produce chemical residues in meat, milk, or by-products derived from animals for human consumption [60]. In this sense, the use of products based on helminthophagous fungi can integrate helminth control systems, contributing to the reduction of the dependence on anthelmintics.

From another perspective, proglottids obtained from adult parasites from the intestinal loops of small ruminants can provide immature eggs. Similar to the study of *Ascaris suum*, the use of these forms led to controversial results, as the eggs of these parasites were protected from fungal action [39]. Considering this, obtaining naturally eliminated proglottids may be more reliable sources for future studies.

It is worth mentioning that biological control is not a substitute for anthelmintics. In situations of health problems, the latter is an excellent tool. The role of biological control is to reduce contamination of pastures and reinfection of animals, thus reducing the need to apply these drugs without affecting their production rates or even improving them [54]. In addition, favoring good management practices for these chemical compounds maintains satisfactory levels of effectiveness for their use [12].

The predatory capacity of *M. expansa* eggs verified in the present study showed that a experimental formulation containing *D. flagrans* and *P. chlamydosporia* fungi has the potential for application in integrated helminth control programs, with *P. chlamydosporia* being a species with ovicidal effect.

## Figures and Tables

**Table 1:** Mean ± standard deviation and standard error (SE) of the number of intact eggs of the respective groups: B1 (total dose of 1 g of the formulation for each animal); B2 (total dose of 1.5 g of the formulation for each animal); B3 (total dose of 1.5 g of placebo for each animal). Three collecting times (24, 48, and 72 h) following ingestion, after 15 days in B.O.D. incubator at 26 °C in the dark.

Mean of Intact Eggs			
Group (n)	24 h	48 h	72 h
B1 (9)	59.0 ^a^ ± 16.3 (SE = 5.4)	42.5 ^a^ ± 14.7 (SE = 4.9)	57.7 ^a^ ± 12.6 (SE = 6.41)
B2 (9)	49.5 ^a^ ± 20.10 (SE = 6.7)	39.1 ^a^ ± 17.9 (SE = 5.9)	46.8 ^a^ ± 18.3 (SE = 6.1)
B3 (9)	115.2 ^b^ ± 21.25 (SE = 10.4)	105.0 ^b^ ± 20.6 (SE = 6.9)	110.4 ^b^ ± 22.47 (SE = 7.49)

Means followed by different lower-case letters in the column show a significant difference of 5%.

**Table 2:** Percentage reduction of *Moniezia expansa* intact eggs using 1 g of the formulation for each animal in group B1 and 1.5 g of the formulation for each animal in group B2 at three collecting times (24, 48, and 72 h) following ingestion, after 15 days in B.O.D. incubator at 26 °C in the dark. The value of group B3 (Control), which received 1.5 g of placebo per animal and their values, were used to calculate the reduction.

Percentage Reduction of Eggs (%)			
Group	24 h	48 h	72 h
B1	49 ^a^	60 ^a^	48 ^a^
B2	57 ^a^	63 ^a^	58 ^a^

Means followed by different lower-case letters in the column show a significant difference of 5%.
